# Deep soils modify environmental consequences of increased nitrogen fertilizer use in intensifying Amazon agriculture

**DOI:** 10.1038/s41598-018-31175-1

**Published:** 2018-09-07

**Authors:** KathiJo Jankowski, Christopher Neill, Eric A. Davidson, Marcia N. Macedo, Ciniro Costa, Gillian L. Galford, Leonardo Maracahipes Santos, Paul Lefebvre, Darlisson Nunes, Carlos E. P. Cerri, Richard McHorney, Christine O’Connell, Michael T. Coe

**Affiliations:** 1 Marine Biological Laboratory, Woods Hole, MA USA; 2 Woods Hole Research Center, Falmouth, MA USA; 3 University of Maryland Center for Environmental Science, Frostberg, MD USA; 4 Imaflora, Piracicaba, SP Brazil; 5 Gund Institute for Environment, University of Vermont, Burlington, VT USA; 6 Instituto de Pesquisa Ambiental da Amazônia, Canarana, MT Brazil; 7 Escola Superior de Agricultura Luiz de Queiroz, Universidade de São Paulo, Piracicaba, SP Brazil; 8 University of Minnesota, St. Paul, MN USA; 9 University of California Berkeley, Berkeley, CA USA; 10 Present Address: USGS Upper Midwest Environmental Sciences Center, La Crosse, WI USA

## Abstract

Agricultural intensification offers potential to grow more food while reducing the conversion of native ecosystems to croplands. However, intensification also risks environmental degradation through emissions of the greenhouse gas nitrous oxide (N_2_O) and nitrate leaching to ground and surface waters. Intensively-managed croplands and nitrogen (N) fertilizer use are expanding rapidly in tropical regions. We quantified fertilizer responses of maize yield, N_2_O emissions, and N leaching in an Amazon soybean-maize double-cropping system on deep, highly-weathered soils in Mato Grosso, Brazil. Application of N fertilizer above 80 kg N ha^−1^ yr^−1^ increased maize yield and N_2_O emissions only slightly. Unlike experiences in temperate regions, leached nitrate accumulated in deep soils with increased fertilizer and conversion to cropping at N fertilization rates >80 kg N ha^−1^, which exceeded maize demand. This raises new questions about the capacity of tropical agricultural soils to store nitrogen, which may determine when and how much nitrogen impacts surface waters.

## Introduction

Rising populations and income levels are expected to increase the global demand for crops by up to 70% by 2050^1–3^. During the last half-century, food demands have largely been met by a combination of expanding croplands and intensifying crop production. In the future, pressures to reduce deforestation and diminishing supplies of arable land will require further cropland intensification such as increased use of fertilizers, greater farm mechanization, irrigation, novel or genetically modified crop varieties, and multiple cropping cycles^1,4^.

Intensive cropping and its associated high fertilizer use have extensive and well-documented environmental consequences^5,6^. Runoff from nitrogen (N) fertilized croplands can impair the quality of fresh and coastal waters, contaminate human drinking water sources, threaten aquatic species, and contribute to hypoxia in coastal zones^7,8^. In addition, use of N fertilizers increases emissions of the powerful greenhouse gas nitrous oxide (N_2_O) from croplands^9–11^.

Although the vast majority of studies of the environmental consequences of fertilizer-intensive cropping are from temperate regions, the most rapid and extensive intensification of crop agriculture now occurs in tropical forest and savanna regions^1,12–14^. Historically, many tropical agricultural systems in South America and Africa have achieved far below their yield potential compared with temperate regions such as the North American Midwest and China^14,15^. This occurred in part because of low N fertilizer use, but that has recently begun to change^11,13,16^. Because much of this tropical cropland expansion and intensification occurs on old, highly weathered soils, the environmental consequences of increasing N fertilizer use may differ markedly from those observed in temperate cropping systems on more recently-developed, less weathered soils^1,4,17^.

The region centered on the Brazilian Amazon state of Mato Grosso encompasses both the Amazon forest and Cerrado (woodland/savanna) biomes, and is among the fastest-growing agricultural frontiers on Earth. Land clearing in the region was predominantly for cattle pasture in the 1980s, but land use shifted to soybean cropping in the 2000s^18,19^. Previous constraints that had excluded soybeans from production in the Amazon were overcome by the development of improved tropical soybean varieties, liming to raise low soil pH and reduce aluminum toxicity, fertilization to increase soil phosphorus (P) and potassium (K), and minimum-tillage to maintain the aggregate structure of relatively high-clay soils^20^. As a result, soybean cropland area increased more than three-fold from 20,000 km^2^ in 1990 to 75,000 km^2^ in 2013^18,21^, and production increased more than ten-fold, from less than 2 million Mt y^−1^ to more than 24 million Mt yr^−1^ ^19^. Expanded production from Mato Grosso fueled Brazil’s rise to become the world’s leading soybean exporter in 2014 (USDA 2017).

In response to high rates of deforestation in the early 2000s, global pressures to change practices resulted in producer-led limits to deforestation through implementation of a “Soy Moratorium” and certification of deforestation-free soybeans^20^. Producers began shifting from single-cropping of soybeans to double-cropping of soybeans with other crops, primarily maize. Maize is planted immediately following soybean harvest, a practice that is possible because of the long, tropical growing season in the region. The transition to double-cropping happened quickly: in 2001, only 500,000 ha of Mato Grosso’s 3.3 million ha of commercial cropland were double-cropped, but by 2011 mechanized agriculture had expanded to 5.8 million ha, of which 2.9 million ha were double cropped^13^. The intensification of existing croplands shifted crop production to previously-cleared land, contributed to the decline of Brazil’s deforestation rate from 14,286 to 4,571 km^−2^ y^−1^ from 2006 to 2010^22^, and made Brazil the third-leading global exporter of maize (USDA 2017). Brazil has made increasing crop production on already-cleared land in the Amazon and Cerrado regions central to its strategy to double agricultural output while further reducing deforestation^22,23^.

This shift from single- to double-cropping has sharply increased N fertilizer use in a region where it has not previously been widely used. Although single-cropping of soybeans requires high inputs of lime, P and K fertilizer, N-fixing soybeans require little or no N fertilizer. In contrast, cultivation of maize as a second crop requires from about 34 to 120 kg N ha^−1^ ^23^. The expansion of maize double-cropping has, therefore, been a major contributor to a 78% increase in Brazil’s N fertilizer use during the last 20 years^16^.

We currently know relatively little about the fate of applied N fertilizers in this or similar tropical regions, but the fate of N in this region could differ substantially compared with observations of similar practices in temperate croplands. First, most of the current expansion of cropping and double-cropping has taken place on Oxisols, which are highly weathered, deep, and very well drained soils despite their high clay content^24^. High soil water infiltrabilty can prevent the soil anoxia that promotes N_2_O production, which is supported by recent evidence of low N_2_O emissions from N-fertilized tropical Oxisols^25,26^. Second, when properly managed, the potential for surface erosion is limited and most runoff reaches streams after passing through deep soil and groundwater flowpaths^24^. This has the potential to buffer surface waters from N applied to fertilized cropland^17,27^. Third, rates of N application on tropical Oxisols (typically <100 kg N ha^−1^) are generally lower than in temperate maize croplands^6,28^, and the common practice of dividing N fertilizer among multiple applications to the same crop may further limit N losses^29^.

Understanding the fate of applied N fertilizer and thresholds for N losses is critical, particularly because the area planted in maize and other fertilized commodity crops is expanding rapidly in Brazil and elsewhere in the tropics. Here, we quantified: (1) how maize yields and maize N use efficiency respond to increased N fertilization rates, and (2) the N_2_O emissions and N leaching losses and potential thresholds associated with increasing N fertilization, and (3) whether N leaching to deep soils increased with conversion to croplands. We conducted a field-level manipulation experiment at Tanguro Ranch, an 800 km^2^ farm in Mato Grosso, Brazil at the center of the region’s predominant soybean-maize double-cropping system. We evaluated maize yields, N_2_O emissions and N leaching from replicated plots treated with five fertilizer levels (0, 80, 120, 160 and 200 kg N ha^−1^) during the maize growing season following soybean harvest. We paired the experiment with field surveys in forests, soybean, and soybean-maize fields to assess the potential for N leaching and storage in deep soils. We used the results of the field experiment to estimate the distribution and magnitude of N_2_O emissions and N leaching across the Amazon-Cerrado cropping region based on soils and the current area of soybean-corn double-cropping. We suggest mechanisms that influence N losses from cropland and approaches to N fertilizer management that would maintain yields but minimize the environmental impacts of this globally-important tropical cropping system.

## Results and Discussion

### Crop yields

Fertilizer application significantly increased maize yield (Table 1), which ranged from 6.4 tons ha^−1^ in fields with no fertilizer addition (control) to 9.1 tons ha^−1^ in fields fertilized with 160 to 200 kg N ha^−1^ (Table 1; linear model with “fertilizer” effect: R^2^ = 0.27, p = 0.005). Yields differed significantly among treatments (Table 1), but this was driven by differences between all treatments and the control (post-hoc Tukey test; p = 0.01). Increasing N fertilizer application beyond 80 kg N ha^−1^ provided no further yield benefits.

**Table 1:** Maize yield and N use efficiency in N fertilizer application treatments.

Fertilizer Input (kg N ha^−1^)	Maize Yield (kg ha^−1^)	Soybean BNF (kg N ha^−1^)	Soybean Harvest Export (kg N ha^−1^)	Maize Aboveground Biomass N (kg N ha^−1^)	Maize Harvest Export N (kg N ha^−1^)	N surplus^a^ (kg N ha^−1^)	N Use Efficiency^b^	N Recovery efficiency (kg kg^−1^)^c^
0	6,400^a^ (400)	217	184	115^a^ (12.4)	69^a^ (5.6)	−36^a^ (5.6)	1.17^a^ (0.03)	N.A.
80	9,100^ab^ (200)	217	184	169^a^ (11.5)	104^ab^ (3.2)	9^b^ (3.2)	0.97^b^ (0.01)	0.68^a^ (0.23)
120	8,400^b^ (900)	217	184	177^a^ (20.3)	95^ab^ (11.6)	58^c^ (12)	0.83^ce^ (0.03)	0.51^a^ (0.18)
160	9,100^b^ (900)	217	184	210^b^ (31.4)	118^b^ (15.5)	75^cd^ (16)	0.80^cd^ (0.04)	0.59^a^ (0.27)
200	9,100^b^ (300)	217	184	198^a^ (9.6)	120^b^ (5.4)	113^d^ (5.4)	0.73^de^ (0.01)	0.42^a^ (0.04)

Treatments with different lettered superscripts differ significantly based on a post-hoc Tukey HSD test. Values in parentheses are standard errors.

^a^Calculated as inputs – exports. ^b^Calculated as exports/inputs. ^c^Calculated as (fertilized export – control export)/fertilizer input.

Maize yield under the typical N application of 80 kg N ha^−1^ yr^−1^ was higher than the 2014–2015 average yield for Mato Grosso of 6.1 tons ha^−1^ yr^−1^ in 2014–2015^30^ and higher than found in other studies in the region, which showed fertilization of 90 to 120 kg N ha^−1^ yr^−1^ produced maximum yields of 5.1 to 7.2 tons ha^−1^ yr^−1^ ^31,32^. Our yield may be higher than the Mato Grosso average because many farmers use less than 80 kg N ha^−1^ yr^−1^ ^16,28^, and higher potential maximum yields at Tanguro Ranch may also result from other agronomic factors or management practices.

### N use efficiency

The N surplus, defined as the difference between all N inputs and harvest outputs during the soybean and maize phases of the cropping cycle^33^, can have multiple fates, such as surface runoff, retention in surface soils, loss to the atmosphere, or leaching to deeper soil layers or groundwater. In our calculations of cropping cycle N surplus, we estimated soybean biological nitrogen fixation (BNF) of 217 kg N ha^−1^ yr^−1^ and harvest removal of 184 kg N ha^−1^ yr^−1^ using previous studies on the same farm^34^. We found a negative N surplus and a N use efficiency (NUE; defined as the ratio of N output in the harvest divided by the sum of N inputs) >1.0 in the unfertilized control. This indicated that the unfertilized maize mined N from the soil (Table 1), which is discussed below. The N surplus increased to 113 kg ha^−1^ yr^−1^ and the NUE decreased to 0.73 as the fertilizer addition increased to 200 kg N ha^−1^ yr^−1^. This NUE was still high compared with global averages of 0.46 for maize single cropping, 0.80 for soybean single cropping, and 0.53 for all crops in Brazil^16,35^. Further, fertilizer recovery efficiency (RE), which is defined as the difference in harvest-N content in fertilized and unfertilized corn divided by the amount of fertilizer used^33^, was highest (0.68) at 80 kg N ha^−1^ and declined with increasing fertilizer application (Table 2). These values were also high compared with the global average for maize (0.37)^35^, although the RE of 0.41 in the 200 kg ha^−1^ treatment approached the global average. The high yields, low N surplus, and high NUE and RE at 80 kg N ha^−1^ yr^−1^ (Table 1) indicated that the fertilization rate used in current practice at Tanguro Ranch was near the optimal rate to meet crop N demand without creating an N surplus that could result in significant N losses.

**Table 2:** Soil extractable nitrate at 0 to 400 cm and soil N_2_O emissions from N fertilizer application treatments.

Treatment	Lysimeter leached (kg N ha^−1^ yr^−1^)	Deep Soil NO_3_^−^ stock (kg N ha^−1^)	NO_3_^−^ leached to deep soil (kg N ha^−1^ yr^−1^)	Maize N_2_O emission (kg N ha^−1^ yr^−1^)	N_2_O emission factor (%)
0	0.21^a^ (0.03)	218^a^ (10.4)	0	0.27^a^ (0.04)	N.A.
80	0.28^a^ (0.10)	285^a^ (49.4)	68.5 (48.9)	0.45^ab^ (0.09)	0.23
120	0.18^a^ (0.04)	328^a^ (38.0)	88.0 (28.7)	0.38^ab^ (0.07)	0.10
160	0.23^a^ (0.04)	382^a^ (61.8)	144 (62.0)	0.58^ab^ (0.07)	0.20
200	0.28^a^ (0.06)	426^a^ (89.0)	148 (75.5)	0.75^b^ (0.17)	0.24

Treatments with different lettered superscripts differ significantly based on a post-hoc Tukey HSD test. Values in parentheses are standard errors.

Our results further suggested that much of the maize N demand was met by BNF during the soybean cropping phase immediately prior to maize planting or from prior years’ N inputs. Unfertilized maize obtained a substantial amount of N fixed by the soybean crop, with a grain N content of 69 kg N ha^−1^ and a total dry biomass N content of 115 kg ha^−1^ (Table 1). Figueira *et al*.^34^ estimated a return of 92 kg N ha^−1^ yr^−1^ from aboveground soy biomass to the soil at Tanguro Ranch, which indicated that BNF during the soybeans phase plus soil organic matter management by conservation tillage could supply a substantial proportion of N demand for the subsequent maize crop. The negative N surplus for the unfertilized control (Table 1) suggested that maize demands exceeded fertilizer and BNF inputs and must have been supported by mineralization of soil organic matter from previous cropping cycles. Aboveground biomass of fertilized maize returned 64 to 88 kg N ha^−1^ yr^−1^ to the soil, much of which could potentially be mineralized and available for crop uptake in subsequent years and may have been available from the previous year’s maize crop. A negative N surplus in unfertilized control plots would not be sustainable indefinitely, however, indicating that some N fertilizer addition would eventually be needed to meet the maize crop demand.

Fertilizer application methods could have also influenced the efficiency of this cropping system. We applied N fertilizer as a split dose, with a small amount (5 kg ha^−1^ as ammonium nitrate) drilled with seeding and a larger dose (75 kg N ha^−1^ as urea) broadcast 21 days later when maize growth and N demand were highest. Had all of the N been applied at the time of planting, the NUE may have been lower and N surplus higher, whereas applying most of the N at a later stage probably better matched plant demand with N supply. This form of split application follows the management practices at Tanguro Ranch, but the extent to which split applications occur across the Amazon-Cerrado region is unknown. Farmers generally have the capacity to vary fertilizer applications in response to soil tests, crop varieties and other conditions. Split applications can increase crop N uptake in other systems^10,29^ and this management practice could potentially increase the efficiency of N fertilizer use by maize and minimize N losses.

### N_2_O Emissions

**Figure 1:**
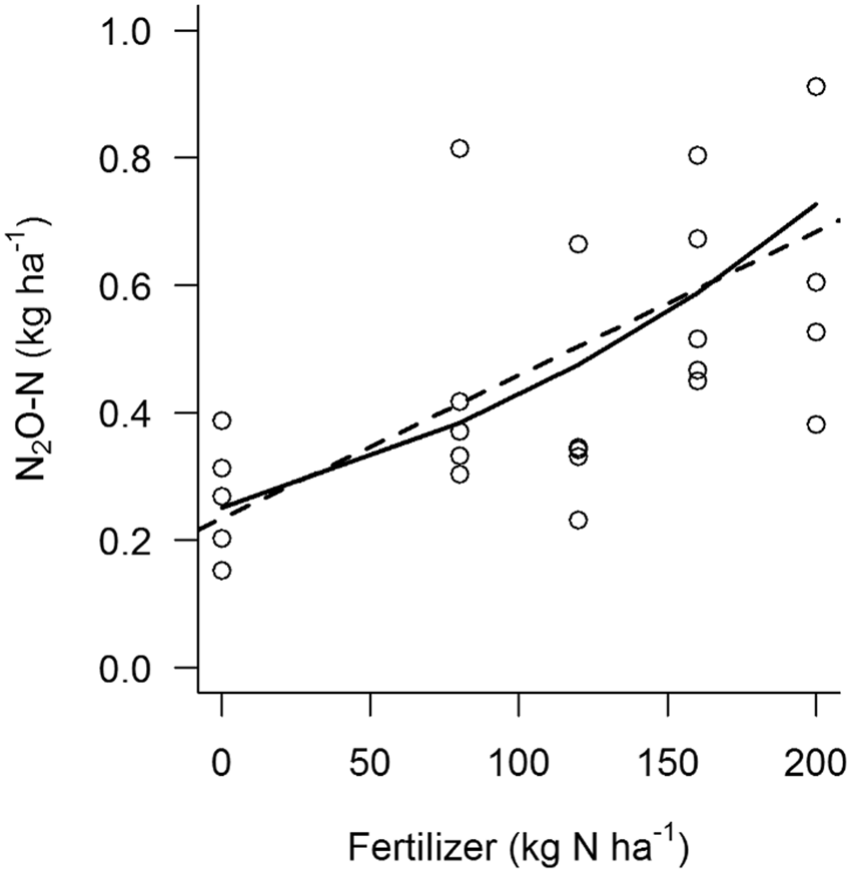
Cumulative N_2_O flux (kg N ha^−1^) from experimental fertilizer treatments during the maize cropping period (30 Jan 30 to 2 Jun 2015). Each point shown at each fertilizer level is a replicate plot within a treatment. Exponential model (“N_2_O_exp” in Table S2, R^2^ = 0.32) fit shown as a solid line and linear model (“N_2_O_lin” in Table S2, R^2^ = 0.32) shown as a dashed line.

Agriculture is estimated to contribute 84% of Brazil’s anthropogenic N_2_O emissions (SEEG, 2017)^36^ and 66% of global N_2_O emissions^37^, making it critical to understand how N_2_O emissions will respond as N use increases on tropical croplands^16,36,38^. Although we found N_2_O emissions increased at higher N fertilization rates (Fig. 1; R^2^ = 0.32, p = 0.002), mean emissions were <0.8 kg N ha^−1^ even at N application of 200 kg N ha^−1^ (Table 2). Emissions factors associated with our 80 to 200 kg N ha^−1^ treatments ranged from 0.09 to 0.26%, and were substantially lower than the 1% estimated by the IPCC (2006; Table 2). Although there was some nonlinearity in the N_2_O response to fertilizer addition, as observed by others^36,39,40^, the linear regression was nearly as good as the exponential model (Table S3).

Our measurements are among the first from intensified croplands in the southern Amazon forest biome and suggest that fluxes are substantially lower than those measured from native evergreen forest of the central Amazon^26^. They were more similar to N_2_O fluxes measured in cultivated systems in Cerrado region^26,41,42^, median fluxes from cultivated systems in Brazil generally (median: 0.80 kg N ha^−1^, range: −0.07 to 4.26)^38^, and maize fertilized at similar levels on Oxisols in another tropical agricultural region of Kenya^25^. In addition, they were substantially lower than fluxes from semi-tropical fertilized croplands and temperate soybean and maize croplands^9,39,40^.

**Figure 2:**
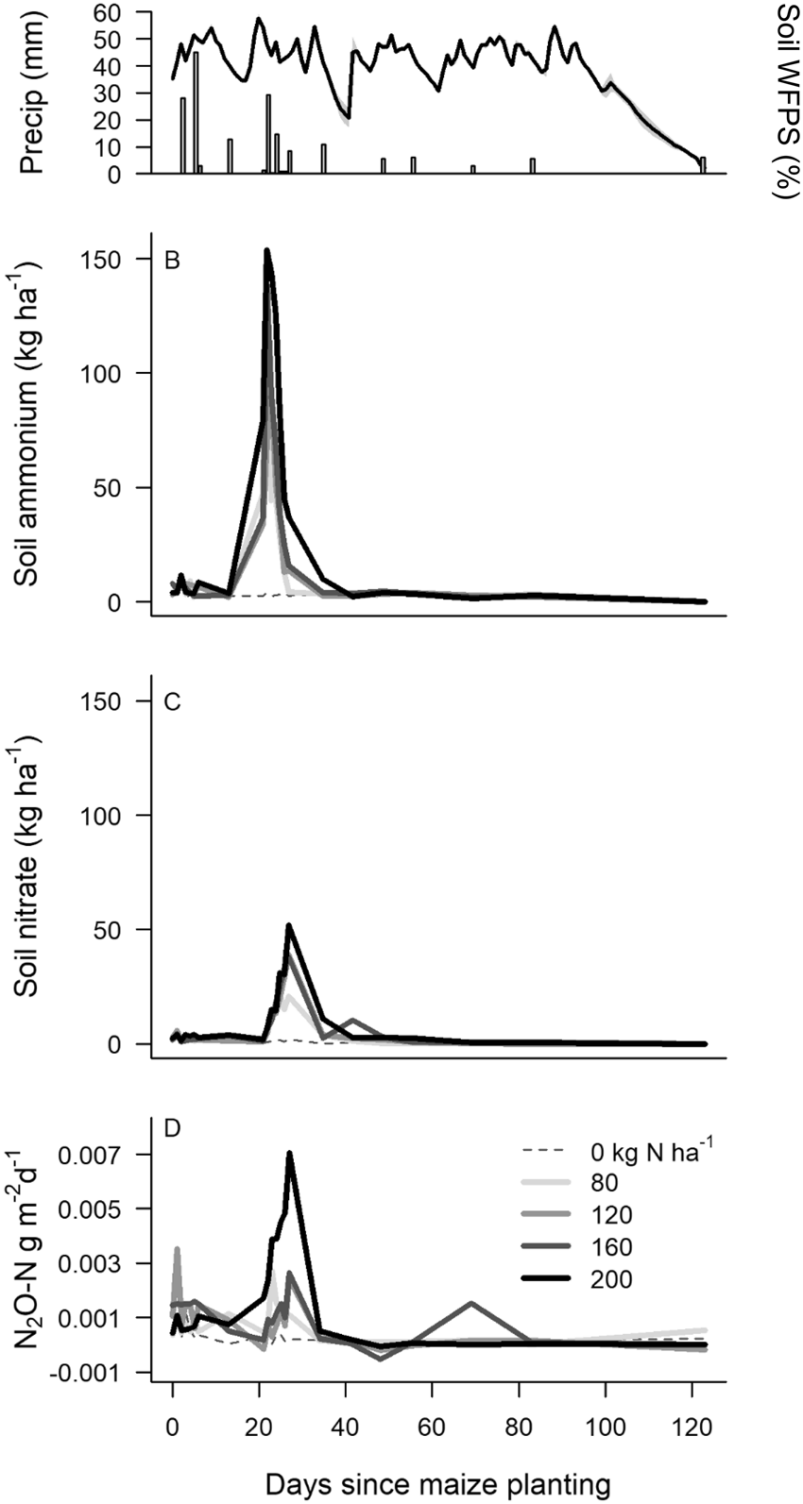
Temporal pattern in: (**A**) precipitation (bars) and water-filled pore space (WFPS; solid line). Gray area represents standard deviation among three soil moisture sensors, (**B**) soil extractable ammonium, (**C**) soil extractable nitrate, and (**D**) N_2_O-N flux during the maize growing season (30 Jan 30 to 2 Jun 2015). The first fertilization of 5 kg N ha^−1^ as ammonium nitrate occurred on 30 Jan 2015 at planting and broadcast fertilization of remaining N as urea was on 20 Feb 2015.

Application of N fertilizer is typically the main driver of N_2_O fluxes from cropping systems^43,44^, which was true in our case as well. Soil extractable ammonium, nitrate and N_2_O fluxes were greatest in the 5 days following maize planting (5 kg N ha^−1^) and in the 15 days following broadcast fertilization (Fig. 2). N_2_O fluxes during the 7 days after broadcast fertilization were higher than at any other point during the cropping cycle, and accounted for 22 to 80% of measured emissions during the maize cropping cycle across all treatments (Fig. 2C, Table 2). We found near-zero fluxes during the soy phase (cumulative flux = 0.01 kg N ha^−1^) and outside of these one-week pulses after fertilizations (Fig. 2). Changes in soil moisture had little effect on N_2_O fluxes outside of these short periods following maize planting and fertilization (Fig. 2).

Low N_2_O fluxes likely resulted from a number of factors. First, much of the added N was taken up by the maize crop and therefore unavailable as a substrate for the nitrification or denitrification that produce N_2_O^45^. Second, these soils have high infiltration capacity and relatively low water-filled pore space, resulting in both high leaching of nitrate below surface soils and oxic conditions that do not favor denitrification-driven production of N_2_O^45^. Lastly, N may have been emitted in forms not measured. Ammonia volatilization is probably low because the soils are naturally acid and liming aims to achieve a pH of 6.5. In addition, N_2_ emissions are likely low in these well-drained soils. However, NO emissions could be significant where nitrification rates are high and soils are well drained^24,45^, as observed in the neighboring Cerrado^41^ and Kenyan Oxisols^46^.

While the time since N application strongly influenced N_2_O flux, we also examined the effects of other factors, such as soil moisture because it can induce the soil anoxia that promotes denitrification. Soil moisture did not have a consistent or statistically significant effect on N_2_O emissions when all sample dates were combined (Table S4). However, soil inorganic N availability and precipitation (or soil moisture) had the greatest effect on N_2_O flux during the weeks following planting and broadcast fertilization (Table S4). After planting, N_2_O flux did not vary significantly among treatments (p > 0.05, ANOVA) and environmental variables explained only 3% of the variation across samples. After broadcast fertilization, soil ammonium and nitrate concentrations explained 31% of the variation in N_2_O flux (Table S4).

### N leaching

N leaching from cropland soils is a primary source of surface water pollution in agricultural regions around the world, including the Mississippi Basin and southeastern China^35,47^. Despite the application of large quantities of N fertilizer and increases in extractable N in the higher N application treatments (Fig. 2), we found very low concentrations of ammonium and nitrate in soil tension lysimeters (Table 2, Fig. S1) and no significant increases in the cumulative nitrate (R^2^ = 0.01; p = 0.28) or ammonium flux (R^2^ = −0.03, p = 061). Because we did not sample soil pore water continuously, it is possible that we missed periods of high N leaching during or immediately after large rainfall events, when soil water storage potential was exceeded. More likely, the lysimeters may not have sampled the portion of the soil solution that accurately reflects leaching in these soils. This could be a function of both the high hydraulic conductivity in these soils that allows water and solutes to move quickly through the soil column in large pores^24^ and the soil pool sampled by tension lysimeters. Hydrologic separation of water used by plants from water that rapidly passes through soils to groundwater is likely widespread^48–50^. This has also been observed on Kenyan Oxisols, where lysimeters fail to sample most soil solution that drains rapidly through the soil macropores^51,52^.

**Figure 3:**
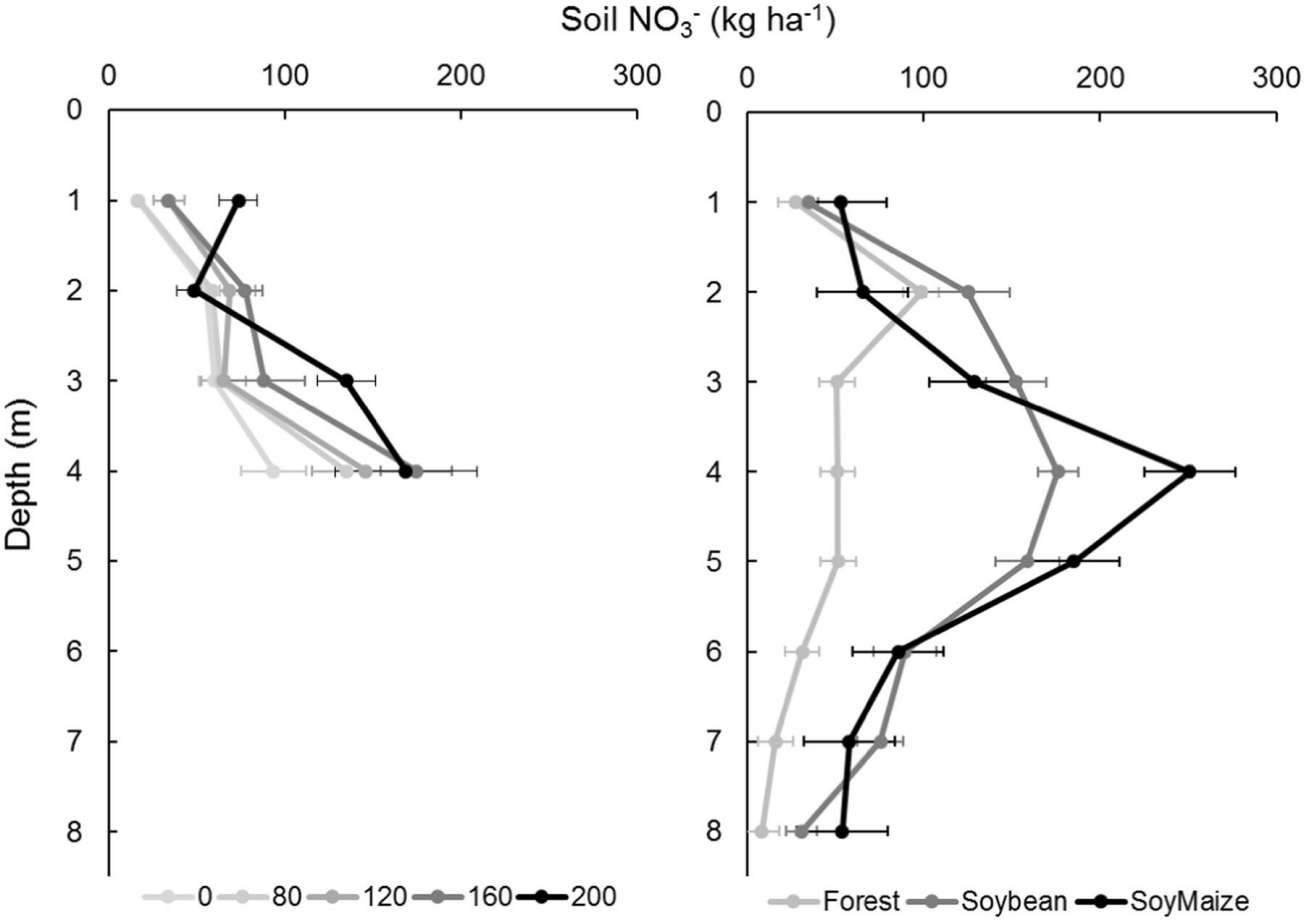
Deep soil extractable nitrate in: (**A**) soils to 400 cm in experimental plots (n = 5 per treatment), and (**B**) soils to 8oo cm in forest (n = 5), soybean (n = 5) and soybean-maize (n = 10) fields. A single site was sampled for each land use. Error bars show standard errors.

To address the potential for inorganic N to leach deeper than the 50 and 150 cm depth of the lysimeters, we collected soils and extracted inorganic N from under our experimental plots to 400 cm depth, after maize senescence and just before maize harvest. We found a high, increasing mass of extractable nitrate with fertilizer application in soils from 0 to 400 cm—from 218 ± 10.4 kg N ha^−1^ (mean ± standard error) in the control to 426 ± 89.0 kg N ha^−1^ in the 200 kg N ha^−1^ treatment (Fig. 3A, Table 2; R^2^ = 0.41, p = 0.006). The mass of extractable ammonium in soils was much lower, and there were no differences with fertilizer application (R^2^ = −0.07, p = 0.87; Table S5, Fig. S2). Most of the mass of nitrate occurred below 100 cm, which lies below rooting depth of soybeans or maize. We calculated the potential N moving to deep soils caused by fertilizer application by subtracting the average extractable nitrate in control treatment from that in each fertilized treatment from 100 to 400 cm depths. The amount of N that moved to below 100 cm in the soil profile increased from 69 ± 49 kg N ha^−1^ in the 80 kg N ha^−1^ treatment to 148 ± 76 kg N ha^−1^ in the 200 kg N ha^−1^ treatment (Table 2). While these values exceed the calculated N surplus shown in Table 1, the error terms are large due to variation in extractable nitrate among soil cores within the same treatment (Table S5). The reported means may overestimate leaching or underestimate N surplus, or both. Although precise accounting is challenging, the important take home points are that: (1) accumulation of deep soil nitrate can account for most or all of the N surplus, and (2) N surplus and leaching to deep soil both increase as the fertilization rate increases, indicating that increasing N fertilizer additions increased the substantial pool of nitrate already stored in deep soils.

We also investigated whether nitrate accumulated at greater depths in croplands (Fig. 3A) and compared deep soil N between croplands and native forests. To do so, we collected soil samples to 800 cm under native forest, single-cropped soybeans and the site of our N fertilizer experiment six months after the final maize harvest. Extractable nitrate was elevated from 200 to 600 cm in all profiles and significantly higher in single-cropped soybeans and double-cropped soybean-maize than in forest (ANOVA: F = 9.49, p = 0.002; Fig. 3B). The total mass of extractable nitrate to 800 cm increased from 335 ± 50.5 kg ha^−1^ in forest to 842 ± 60.0 kg ha^−1^ in single-cropped soybeans and 880 ± 96.4 kg ha^−1^ in double-cropped soybean-maize. Cropland stored N significantly exceeded that in forest soils (Tukey’s HSD test: soybean-forest: p = 0.009; maize-forest: p = 0.002). Extractable ammonium showed no pattern with depth and did not differ among land uses (Fig. S2, Table S5; ANOVA F = 0.757, p = 0.484).

Although deep extractable nitrate was higher in croplands than forests, it did not differ between the soybean and soybean-maize croplands. This suggests that most of the increase in extractable nitrate in deep soils occurred during previous 10 years of soybean cropping, with no detectable increase thus far under the double-cropping system, which had been practiced on that field for only two years. Storage of extractable nitrate in deep soils has also been observed in central Amazon forests^53^ and agricultural Oxisols of Kenya^51^, suggesting that these soils have anion exchange capacity that allows them to hold large quantities of nitrate, in contrast to less weathered temperate soils in which nitrate is typically highly mobile^54–56^. High nitrate pools at several meters depth after logging, in perennial crop culture^53,57^, and after the addition of added ^15^N-labeled fertilizer^58^ have been observed in similar soils in the adjacent Cerrado region of Brazil. Deep soil nitrate storage in the croplands of Tanguro Ranch is also consistent with low streamwater concentrations of nitrate, which have not increased with land use transitions from forest to single-cropped soybeans^27^.

We currently have little understanding of the capacity of these soils to store or remove N over the long term. In native seasonally-dry Amazon evergreen forest, trees have deep roots that allow them to access water during a several months-long dry season^59^, which may allow forests to access and recycle nitrate to the surface. In contrast, the majority of nitrate in cropland soils accumulated below the rooting zone of more shallowly-rooted soybean and maize crops, and, thus, no mechanism exists to recycle this N to the soil surface. Denitrification in deep soils or in groundwater is another plausible but unknown fate of the deep soil nitrate. The capacity of deep soils to denitrify or to continue to accumulate nitrate under intensive cropping will likely determine the time over which this cropping can continue in the absence of significant breakthrough of N runoff to surface waters.

### Regional implications

**Figure 4:**
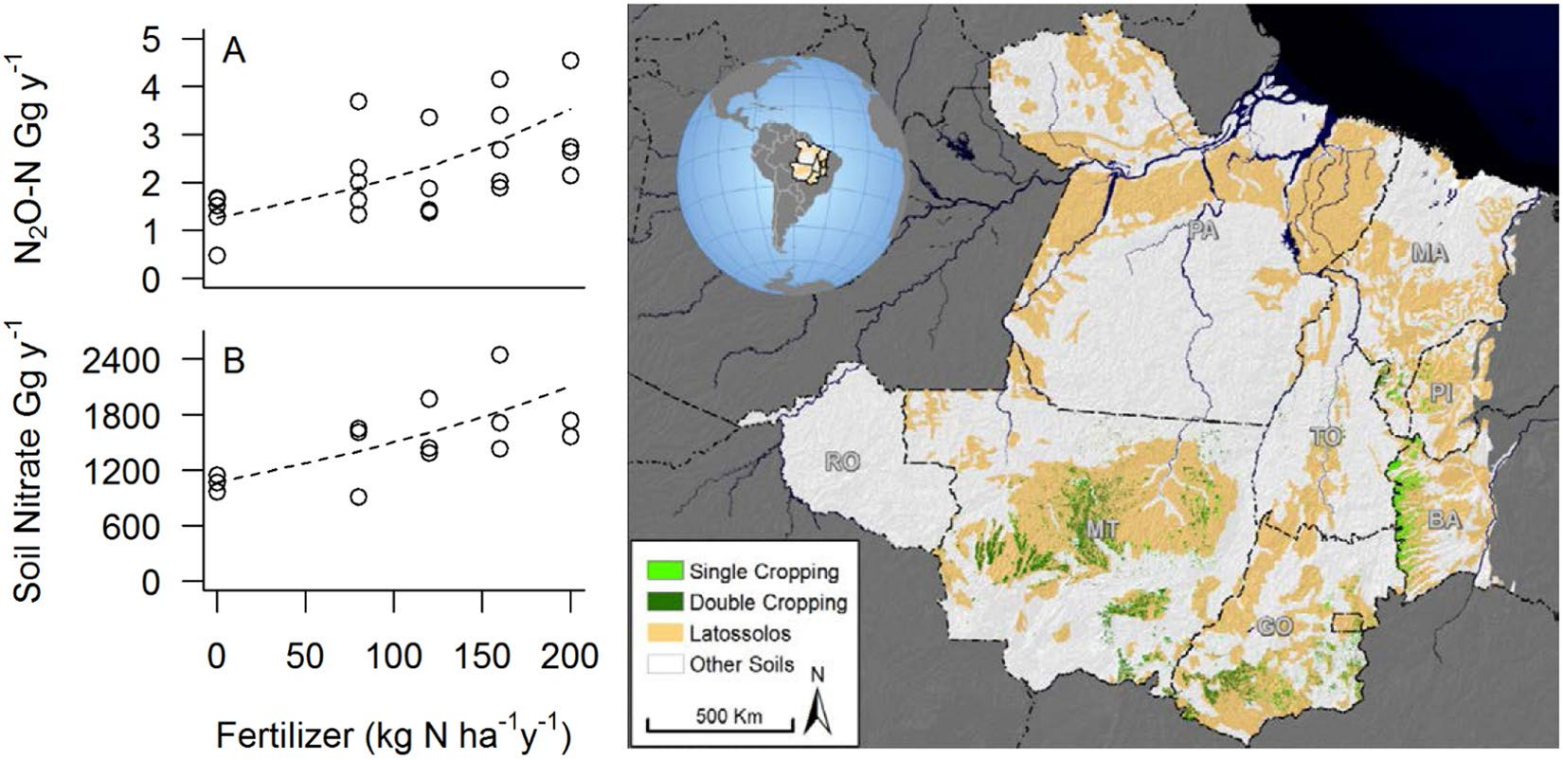
Estimates of potential: (**A**) N_2_O-N flux, and (**B**) Deep soil extractable nitrate if scaled to currently double-cropped area determined by Spera *et al*.^18^ underlain by Oxisols (Latossolos in the Brazilian classification). Nitrate that moved into deep soils was estimated as the nitrate measured at 100 to 400 cm in the 80, 120, 160, and 200 kg N ha^−1^ treatments after the N fertilization experiment minus the nitrate at the same depth in the control treatment. Plotted line is best model fit to scaled data based on Akaike information criterion values adjusted for small samples (AIC_c_) shown in Table S2. Map and globe inset were created in ArcMap, v10.5.

Although we measured relatively low N_2_O fluxes and N leaching, the sheer geographic scale of soybean-maize double cropping in the region could have implications for both greenhouse gas emissions and N leaching into waterways. We investigated the potential N_2_O emissions and N leaching from increasing N fertilization across areas that are currently double-cropped and on similar soils. As of 2015, 2.3 million ha of the region’s soybeans (total soybean = 3.4 million ha) and 4.9 million ha of its soybean-maize double-cropping (total soy-maize = 6.1 million ha^19^, Fig. 4) occurred on Oxisols. If our measured N_2_O emissions represent those in the region, this would translate into 2.2 Gg of N_2_O-N emissions from soybean-maize double cropped lands fertilized at 80 kg N ha^−1^ or 3.7 Gg of N_2_O-N emissions if fertilized at 200 kg N ha^−1^. This is the equivalent of 8.8 to 14.7% of the total direct N_2_O-N emissions from N fertilizer application in Brazil (29.4 Gg N_2_O-N; MCTI, 2015; SEEG, 2015).

In addition, under current fertilizer application rates of approximately 80 kg N ha^−1^, we estimated that 335 Gg of nitrate would be lost annually to deep soils across the current area of double-cropping. This N loss to deep soils would increase to 725 Gg if N fertilizer application increased to 200 kg N ha^−1^. Although there is little evidence that this N has reached surface waters, this amount of stored N is large and equivalent to 33 to 70% of the 2016 N load to the Gulf of Mexico from the Mississippi Basin of 1000 Gg^60^. The scaling of our results from a single location to the region certainly includes a large degree of uncertainty, However, the large potential magnitude of estimated regional increases in N_2_O emissions and N leaching indicate that these concerns merit more attention and study. If Tanguro Ranch has above average NUE for the region, regional N losses to the environment could be underestimated by our extrapolation. Future work should investigate how these processes respond across wider gradients of precipitation and field management practices to better constrain the variation across the region. The fate of this stored nitrate could have important implications for N loading to fresh and marine waters of the Amazon Basin and indirect emissions of N_2_O^44^ if it is ever mobilized to groundwater and streams.

Brazil’s Amazon agriculture is undergoing major expansion and intensification, with implications for both agricultural productivity and environmental sustainability. Our study quantified some of the tradeoffs implicit in intensifying agriculture through soy-maize double-cropping by evaluating the influence of additional fertilizer on maize yield and two of its key environmental consequences—emissions of the greenhouse gas N_2_O and N losses. Our results suggest that further increases in fertilizer application will not greatly increase maize yields or emissions of N_2_O, but could add substantially to a large pool of nitrate stored in deep soils. This nitrate accumulation in soil profiles currently appears to limit hydrologic N losses, but because most N accumulation occurs below the crop rooting depth, further nitrate accumulation is likely with more years of cropping. The nitrate storage capacity of these soils is currently unknown, but if exceeded, could cause nitrate breakthrough to surface waters in this and other intensifying agricultural regions occurring on similar soils around the tropics.

## Methods

In 2015, Tanguro Ranch had 16,896 ha of single-cropped soybeans, 16,551 ha of double-cropped soybeans and maize, and the remainder of land in forest. Mean annual temperature is 27 °C and mean annual precipitation is 1,800 mm yr^−1^, most of which falls from November to April when crops are grown (1987–2010 mean; Tanguro Ranch, unpublished data). Soils on are medium textured, highly weathered, base-poor ustic Oxisols (Latossolo vermelho-amarelo distrófico in the Brazilian classification; IBGE-EMBRAPA 2001). Soils and the depth to the groundwater table are deep (10–30 m; C. Neill, unpublished data), very well drained on plateaus^24^, and have a mean soil texture of 55% sand, 2% silt, and 43% clay across all land uses^61^. Soil pH under native forest is 3.9 and soil pH in established croplands is 6.0^61^. See Dias *et al*.^62^ for description of soil hydraulic properties and Riskin *et al*.^61^ and Figueria^63^ for data on soil P and other chemical content (e.g., K^+^, Mg^2+^, ECEC) at Tanguro Ranch.

We established a fertilizer manipulation experiment in January 2015 in a ~1 km^2^ field (Field 48) that had been double-cropped since 2014 (Fig. S3). We tested five levels of N fertilization (0, 80, 120, 160 and 200 kg N ha^−1^) in five replicate plots (6 × 7.5 m, including a 0.5 m unfertilized buffer around the perimeter) with treatments randomly assigned within blocks. We used the same form of N (ammonium-nitrate and urea) and fertilized at the same time as Tanguro Ranch. Ranch managers planted soybeans on Field 48 when rains started in early November 2014, harvested them on 29 Jan 2015, and planted maize a day after soybean harvest (30 Jan 2015). Five kg N ammonium nitrate drilled into the row at seeding in all treatments except the 0 kg N ha^−1^ control had. We broadcast fertilized our experimental plots with the appropriate levels of urea 21 days after planting. At the end of the maize growing season on 2 June 2015, we harvested 10 whole maize plants per plot (n = 50 per N fertilizer level) to calculate yields and maize N content. We separated each plant into aboveground and belowground biomass, cobs, and kernels. Plant components were pooled, weighed wet, dried for 24 hours at 65 °C, and re-weighed to estimate total biomass and water content. A composite subsample of each plant component per plot (n = 5) was ground and analyzed for total C and N content on an elemental analyzer, and dried and weighed for gravimetric water content (Fig. S6).

To estimate crop nitrogen balances, we used partial nutrient balance NUE and crop recovery efficiency (RE). The partial nutrient balance NUE, also known as output/input ratio, was defined as the annual sum of harvest outputs of N in soybeans and maize grain divided by the sum of fertilizer and biological N fixation (BNF) inputs^33,35^. We assumed that all fields had the same input of BNF by soybeans (217 kg N ha^−1^), the same total soybean N content (276 kg N ha^−1^), and same total export of N via the soybean harvest (184 kg N ha^−1^) based on ^13^C and ^15^N isotopic analysis and mass balance estimates done by Figueira *et al*.^34^. Because we had a no-fertilizer control treatment, we could also calculate the crop recovery efficiency (RE), which is a less commonly used metric of NUE, defined as (U – U_0_)/F, where U and U_0_ are the total N uptake by the aboveground crop biomass of the fertilized and unfertilized plots, respectively, and F is the amount of fertilizer added^29,33^.

We sampled N_2_O fluxes four times during the soybean phase (3 Dec 2014 to 29 Jan 2015) daily for six days immediately following maize planting (30 Jan to 5 Feb 2015), once on 12 Feb, daily for seven days immediately following broadcast fertilization (20–26 Feb), and at weekly or bimonthly intervals from 5 March until harvest on 2 June. We sampled using static chambers^64,65^ at four times (0, 15, 30 and 45 minutes) per chamber, and we fit a linear model to these four time points to estimate fluxes. We removed chamber flux values for which the R^2^ of the linear model fit was less than 0.90. N_2_O emissions factors for each individual treatment were calculated (i) using annual cumulative N_2_O emissions as follows: (N_2_O_TRT,i_ − N_2_O_CTL_)/N_Fert,i_. To calculate cumulative fluxes for outside the maize planting period of our experiment, we used measurements taken from the soybean period prior to maize planting on the same field (cumulative flux = 0.01 kg N ha^−1^) and an average estimate from double-cropped fields at this site taken from O’Connell (2015; −0.01 kg N ha^−1^). See the Supplemental Methods for more detailed description of chamber construction, gas sampling methods, and calculation of cumulative N_2_O fluxes.

We collected one surface soil sample for analysis of exchangeable ammonium and nitrate at 0 to 10 cm depth within 100 cm of the gas sampling chamber in each plot at the time of each N_2_O measurement. We estimated leaching of inorganic N from our experimental treatments by sampling soil solution at 50 and 150 cm using tension lysimeters, which were installed the day of maize planting and sampled 10 times during the experiment at corresponding times to N_2_O measurements (see Supplemental Methods for details of lysimeter construction and N load calculations). We also sampled soil profiles to 400 cm under our experiment at the end of the growing season in June 2015 by hand auguring to 200 cm in all experimental plots (n = 5 per treatment) and to 400 cm in a subset (n = 3 per treatment). To evaluate patterns in N storage below 400 cm and across land uses, we also sampled soils in a well-studied intact forest plot (0.5 km^2^, n = 5), in one large soybean field (1 km^2^, n = 5) and in Field 48 (n = 10) to 800 cm.

We used the results of our fertilizer manipulation experiment, estimates of the area of single- and double-cropping in the Amazon-Cerrado cropland frontier region, and soil maps to estimate N_2_O fluxes and N leaching from current croplands on similar soils. To estimate N_2_O flux and leaching losses from current croplands, we combined data on areas of single- and double-cropping calculated originally from Spera *et al*.^13^ and Spera *et al*.^18^ with estimated areas of single cropping and double-cropping across the 1.97 million km^2^ Amazon-Cerrado region that stretches from Rondônia to the Maranhão-Tocantines-Piauí-Bahia (MaToPiBa). We used the intersection of the area of latosolos (IGBE-EMBRAPA 2001) with the total area of 2015 of single and double cropping to scale our annual rates of N_2_O flux and N leaching from Tanguro.

To evaluate differences in crop yield, N_2_O fluxes, and N leaching among experimental treatments we evaluated whether the increase with treatment was linear, exponential, polynomial, or took the form of a Michaelis-Menten or logistic curve (see Supplemental methods for model specifications and Figs S4 and S5 for model fits). We compared among these models using AICc^66^ which showed that in most cases, linear or exponential models explained the patterns in the data equally well (Table S3, Figs S4 and S5). We report the model statistics from the linear model (R^2^ and p-value) in the text. We also used an ANOVA followed by a post-hoc Tukey HSD test to investigate whether there were significant differences among individual treatments. To test the effects of chemical and physical variables on our measured daily N_2_O fluxes, we used linear mixed effects models and compared them using AIC_c_^66^. We report R^2^ for all model fits using marginal R^2^ and conditional R^2^ ^67^. Mixed models were performed using the “lme4” package^68^. All analyses were done in R^69^. See Supplemental Methods for details of model development and comparisons.

## Electronic supplementary material

Supplemental Material

## Data Availability

The datasets generated during and/or analysed during the current study are available from the corresponding author on reasonable request.
