# Supplementary material for: Deep soils modify environmental consequences of increased nitrogen fertilizer use in intensifying Amazon agriculture
Source: Sci Rep. 2018 Sep 7;8:13478. doi: 10.1038/s41598-018-31175-1 (PMC6128839; doi:10.1038/s41598-018-31175-1)
Supplement: Supplementary file 1 — Supplemental Material [file 41598_2018_31175_MOESM1_ESM.docx]

**Deep soils modify environmental consequences of increased nitrogen fertilizer use in intensifying Amazon agriculture**

Jankowski, KathiJo, Christopher Neill, Eric A. Davidson, Marcia N. Macedo, Ciniro Costa Junior, Gillian L. Galford, Leonardo Maracahipes Santos, Paul Lefebvre, Darlisson Nunes, Carlos E. P. Cerri, Richard McHorney, Christine O’Connell, and Michael T. Coe

**Supplemental Material**

**Farming practices at Tanguro Ranch**. Forests at Tanguro were originally cleared for cattle pasture in the early 1980s, converted to single-cropped soybeans between 2003 and 2008 and to double-cropped with maize on some fields beginning in 2010. When pastures were converted to soybean cropland, woody vegetation was burned, soil was tilled to 50 cm, and lime was incorporated. Soils were then disked to 30 to 40 cm for one to two years and subsequently managed by minimum-tillage. We obtained information on fertilizer practices from Tanguro Ranch managers. In typical cropping practice at Tanguro Ranch, lime is added at approximately 1500 kg ha^-1^ every other year. In single-cropped soybean or during the soybean phase of soybean-maize double-cropping, approximately 50 kg ha^-1^ P (as rock phosphate, single super phosphate, or triple super phosphate) and approximately 70 kg ha^-1^ potassium (as KCl) are added annually (Riskin et al. 2013 and Tanguro Ranch). In the maize phase of double-cropped soybeans and corn, 5 kg N ha^-1^ (as ammonium nitrate) are incorporated in the row during corn seeding and an additional 65 to 75 kg N ha^-1^ (as urea) are broadcast approximately 21 days after planting. Approximately 9 kg P/ha (as single or triple superphosphate) and 31 kg ha^-1^ P (as KCl) are also broadcast 21 days after maize planting.

**Experimental setup.** All treatments had five replicate plots randomly assigned within blocks. Individual plots were 6 × 7.5 m, including a 0.5 m unfertilized buffer around the perimeter to avoid cross-contamination (Figure S4). Tanguro Ranch staff seeded maize into all experimental plots the day after soybean harvest (Figure S4) with a mechanical planter. All plots were within one pass of the mechanical planter and received 5 kg N ha^-1^ as ammonium nitrate at planting. Control plots were within an adjacent pass of the mechanical planter and were seeded but received no fertilizer. Levels of P and K were constant across treatments and matched farm practice. All plots received 9 kg P ha^-1^ (as triple superphosphate) and 31 kg K ha^-1^ (as potassium chloride). We hand broadcasted additional N (as urea) 21 days after planting (20 Feb 2015) to bring the total N applications in the treatment plots to 80, 120, 160 and 200 kg N ha^-1^ in the appropriate plots.

**N_2_O fluxes**. We used static chambers to sample N_2_O from soils. Chamber bottoms were made by cutting the bottom of stainless steel restaurant “steamtable” pans (20 gauge, 53 cm long x 32 cm wide x 12 cm deep; Venterea et al. 2005). Chamber tops were made of an additional pan with reflective aluminum insulation wrap to prevent large temperature changes, sealing the bottom edges with rubber weather stripping (ethylene propylene diene tetrapolymer), and cutting a small hole into which we installed a sampling port fitted with a rubber septum. Metal binder clips fastened the chamber top and bottom together during sampling. A vent tube (0.64 cm diameter, 20 cm long, stainless steel) was inserted near the bottom edge of the chamber top to allow pressure to equilibrate within the chamber. Chamber bottoms were permanently installed into the soil the day the maize was planted. Fluxes were measured between 8:00 and 12:00 h local time. To sample, we closed the chambers and collected 12 mL gas samples with a plastic syringe at 0, 15, 30 and 45 minutes. Samples were injected into 9 mL pre-evacuated serum vials sealed with butyl rubber septa (Grace Davidson). Samples were kept cool and transported by bus to Piricicaba, São Paulo for analysis by ECD gas chromatography on a Shimadzu greenhouse gas analyzer.

Standard curves and system calibration were done using analytical grade standards (Scott Specialty Gases, Plumsteadville, PA). Gas fluxes were calculated according to Hutchinson and Mosier (1981). The rate of change of concentration of N_2_O over time at each chamber was used in combination with the chamber volume and soil surface area within the chamber to convert chamber gas concentrations first, from parts per million (ppm, determined by GC analysis) to mass per chamber volume using the Ideal Gas Law and, second, to units of mass per unit area (mg N-N_2_O m^-2^).

To calculate cumulative fluxes over the growing season, we linearly interpolated measured fluxes between sampling time points. For the entire soybean and maize cropping season, we summed these interpolated daily fluxes between December 2014 and 2 June 2015. For the maize growing period, we summed fluxes between maize planting (30 Jan) and maize harvest (2 June). To calculate annual fluxes and emissions factors, we combined the cumulative growing season flux with an average dry season value measured in fertilized maize fields by O'Connell (2015) from (3 Jun to 30 Nov from O'Connell (2015) at Tanguro scaled across the dry season fallow period (Table S2).

**Soil N***.* At the time of field collection, we collected soils and immediately extracted them with 2N KCl for 24 h. After extraction, soils were filtered (Whatman No. 1) and extracts preserved by freezing. Extracts were then analyzed for NH_4_^+^ and NO_3_^-^ by automated flow injection analysis (Lachat Quickchem 8500) at the Marine Biological Laboratory (Woods Hole, MA). We determined gravimetric water content of each soil sample by drying a subsample to 105 ^o^C and reweighing.

We sampled soils below all experimental plots (n=5) to 200 cm at the following depths: 0-10 cm, 10-50 cm, 50-100 cm, 100-150 cm and 150-200 cm and to 400 cm in a subset of plots (n=3 per treatment) at 200-250 cm, 250-300 cm and 300-400 cm. To quantify the exchangeable N present in soil profiles, we summed the total ammonium and nitrate present in the soil column in the plots in which we sampled to 400 cm (n=3 per treatment), accounting for the volume and bulk density of each sampled soil layer (63). To calculate N leached, we calculated the difference between the total inorganic N in each treatment plot and the average of all control plots in soils from 100 to 400 cm (below the crop rooting zone). To compare deep soil N storage among land uses and at greater depths, we sampled five locations (20 m apart) at each one forest, one soybean and one soy-maize site to 800cm at the following depths: 0-10 cm, 10-50 cm, 50-100 cm, 100-150 cm, 150-200c m, 200-300 cm, 300-400 cm, 400-500 cm, 500-600 cm, 600-700 cm, 700-800 cm. Total soil N stocks were calculated using bulk density measured to 800 cm from existing 1000 cm deep soil pits in the forest, the soybean field, and in a soybean-maize field in the same topographic position ~5 km from Field 48.

**Lysimeter sampling and nitrogen leaching.** Lysimeters were constructed from ceramic cups (5.7 cm diameter, 1 barr, Soilmoisture Equipment Corp.) attached to plastic tubing. Samples were collected by placing a vacuum on the lysimeters and collecting water the following day. Samples were stored on ice and returned to a field laboratory. Samples for analysis of ammonium and nitrate were preserved with Thymol and frozen. Samples for analysis of total dissolved N were preserved with concentrated HCl and frozen. We measured ammonium and nitrate, at the University of São Paulo in Piracicaba using inductively coupled plasma optical emission spectroscopy (Ultima 2, Horiba Jobin Yvon, São Paulo, Brazil) and we measured total N by automated high temperature combustion (TOC-CPH, Shimadzu, Columbia, MD).

We calculated total N leached by using concentrations in the 150 cm lysimeters multiplied by recharge estimates determined from a field water balance calculated as the difference between locally-measured rainfall and calculated evapotranspiration (29). We used daily precipitation data from rain collectors installed at the field site. ET was estimated to be 130 mm/month for maize crops grown in this region using the Global Terrestrial ET Data Set (MOD16), which models monthly ET using satellite data from the Moderate Resolution Imaging Spectroradiometer (MODIS) and an algorithm based on the Penman-Monteith equation (70).

**Scaling.** We used data on single- and double-cropping and soils from the region that stretches from Rondônia to the Maranhão-Tocantines-Piauí-Bahia (MaToPiBa). Cropping data were updated to the year of our field experiment in 2015 (Spera et al. 2016, S. Spera, personal communication). Areas of maize and cotton were combined into total double-cropped area because maize is more than 90% of second cropping (13). We then estimated the total area of the level, deep and highly weathered Oxisols that are generally similar to the soils at Tanguro Ranch from the area of the Latossolo soil class using national soils maps (IBGE-EMBRAPA 2001).

**Data Analysis.** We compared among the following models when evaluating how yield, N_2_O and leaching responded to fertilizer addition:

(1) Linear model: $y_{i}=\beta_{1}x_{i}+\beta_{0}+\varepsilon$

(2) Polynomial model: $y_{i}= \beta_{1}x_{i}+ \beta_{2}x_{i}^{2}+ \beta_{0}+ \varepsilon$

(3) Exponential model: $y_{i}= e^{\alpha+\beta x_{i}}$, *with α = initial value and β = rate of increase*

(4) Logistic model: $y_{i}= \frac{L}{1+ X_{m}e^{kx_{i}}}$ , *with L = asymptote, X_m_ = midpoint, and k = slope*

(5) Michaelis-Menten model: $y_{i}= \frac{V_{max} x_{i}}{k_{m}+ x_{i}}$ , *with V_max_ = maximum value, k_m_ = half saturation constant*

* *x* = Treatment level, *y* = response variable, *i* = observation number

**N_2_O Flux model comparisons.** We used linear mixed effects models to compare among local physical-chemical drivers of N_2_O fluxes. We first tested among models with differing random effects structures: 1) a model with no random effects, 2) a model with a random effect of “Plot” on the intercept, and 3) a model with a random effect of “Plot” and “Block” on the intercept. Model comparison using AICc showed that adding a random effect of “Block” did not improve the model, therefore we fit models with only a random effect of “Plot”, which accounted for repeat samples taken from individual plots. We then compared among models with different fixed effects, which included ammonium, nitrate, soil temperature (Temp), soil moisture (SoilH_2_O), and precipitation over the previous 24 hours. We centered the variables using z-scores to standardize among different measurement scales. We evaluated whether linear model fits conformed to assumptions of normality by visually inspecting the distribution of model residuals for heteroscedasticity, and found that they were normally distributed.

**References**

Hutchinson, G.L. and Mosier, A.R. (1981) Improved soil cover method for field measurement of nitrous oxide fluxes. Soil Science Society of America Journal, 45, 311- 316. doi:10.2136/sssaj1981.03615995004500020017x

O’Connell C (2015) Ecological tradeoffs to an agricultural Amazonia: Investigating the effects of increased agricultural production on Amazonia’s contribution to global climate and nitrogen cycling. Dissertation (University of Minnesota). Available at: <http://hdl.handle.net/11299/177116>.

Riskin SH, et al. (2013) The fate of phosphorus fertilizer in Amazon soya bean fields. Philos Trans R Soc B Biol Sci 368(1619):20120154–20120154.

Spera SA, Galford GL, Coe MT, Macedo MN, Mustard JF (2016) Land-use change affects water recycling in Brazil’s last agricultural frontier. Glob Change Biol 22(10):3405–3413.

Venterea RT, Burger M, Spokas KA (2005) Nitrogen Oxide and Methane Emissions under Varying Tillage and Fertilizer Management. Journal of Environment Quality,

34, 1467.

**Table S1. Biomass and nitrogen content of harvested maize.**

**Table S2.** Cumulative N_2_O fluxes from soybeans, maize, and the dry season. See text for date ranges. Emissions factors are calculated based on total annual fluxes.

**Table S3.** Comparison of models evaluating the response of maize yield, cumulative N_2_O flux, and deep soil N data from 0 to 400 cm and from 100 to 400 cm to increasing fertilizer application (“lin” = linear, “exp” = exponential, “poly” = polynomial, “mm” = Michaelis-Menten, and log = logistic). R^2^ values shown for linear and polynomial models and RMSE shown for all models.

| **Model** | **n** | **k** | **AICc** | **ΔAICc** | **RMSE** | **R^2^** |
| --- | --- | --- | --- | --- | --- | --- |
| **N2O Flux** |  |  |  |  |  |  |
| N2O_exp | 25 | 2 | -1.6 | 0.0 | 0.21 | 0.32 |
| N2O_lin | 25 | 2 | -0.6 | 1.0 | 0.21 | 0.32 |
| N2O_poly | 25 | 3 | 1.0 | 2.5 | 0.21 | 0.31 |
| N2O_log | 25 | 3 | 6.2 | 7.7 | 0.23 | 0.08 |
| N2O_mm | 25 | 2 | 6.4 | 8.0 | 0.24 | 0.07 |
| **Maize Yield** |  |  |  |  |  |  |
| yield_poly | 25 | 2 | 90.6 | 0.0 | 1.25 | 0.32 |
| yield_lin | 25 | 2 | 91.4 | 0.8 | 1.32 | 0.27 |
| yield_log | 25 | 3 | 91.7 | 1.2 | 1.24 | 0.28 |
| yield_exp | 25 | 2 | 91.9 | 1.3 | 1.33 | 0.22 |
| yield_mm | 25 | 3 | 135.8 | 45.3 | 3.22 | -3.4 |
| **Soil N Storage (0-4m)** | |  |  |  |  |  |
| NO3_exp | 25 | 2 | 180.3 | 0.0 | 79.4 | 0.41 |
| NO3_lin | 25 | 2 | 180.4 | 0.1 | 79.7 | 0.41 |
| NO3_poly | 25 | 3 | 182.9 | 2.6 | 79.4 | 0.37 |
| NO3_log | 25 | 3 | 190.1 | 9.8 | 107.8 | -0.14 |
| NO3_mm | 25 | 2 | 194.1 | 13.8 | 125.6 | -0.48 |
| NH4_lin | 25 | 2 | 113.0 | 0.0 | 8.4 | -0.07 |
| NH4_exp | 25 | 2 | 113.0 | 0.0 | 8.4 | -0.09 |
| NH4_log | 25 | 3 | 113.7 | 0.7 | 8.4 | -0.59 |
| NH4_poly | 25 | 3 | 113.9 | 0.9 | 8 | -0.04 |
| NH4_mm | 25 | 3 |  |  |  |  |
| **Soil N Leaching (1-4m)** | | |  |  |  |  |
| NO3_lin | 20 | 2 | 146.1 | 0.0 |  | 0.06 |
| NO3_mm | 20 | 2 | 146.1 | 0.0 |  | 0.10 |
| NO3_exp | 20 | 2 | 148.4 | 2.3 |  | -0.06 |
| NO3_log | 20 | 3 | 148.8 | 2.7 |  | -0.06 |
| NO3_poly | 20 | 3 | 148.9 | 2.8 |  | -0.04 |
| NH4_lin | 20 | 2 | 92.1 | 0.0 |  | -0.03 |
| NH4_log | 20 | 3 | 93.7 | 1.6 |  | -0.15 |
| NH4_poly | 20 | 3 | 93.8 | 1.7 |  | -0.05 |
| NH4_exp | 20 | 2 |  |  |  |  |
| NH4_mm | 20 | 3 |  |  |  |  |

**Table S4.** Model comparison of environmental controls on daily N_2_O fluxes during three time periods 1) planting, 2) fertilization, and 3) over the entire maize growing season. Precip = precipitation in previous 24-hour period, SoilH_2_O = soil moisture, & Temp = soil temperature. SoilH_2_O and Temp are averages over the N_2_O sampling time period 08:00-12:00. Only models within 30 AIC_c_ units of the best model are shown.

| **Model** | **n** | **k** | **AICc** | **ΔAICc** | **R^2^,_m_** | **R^2^,_c_** |
| --- | --- | --- | --- | --- | --- | --- |
| **PLANTING** |  |  |  |  |  |  |
| SoilH_2_O | 166 | 4 | 439.45 | 0.00 | 0 | 0.41 |
| NH_4_^+^  + SoilH_2_O | 166 | 5 | 444.88 | 5.43 | 0 | 0.42 |
| NH_4_^+^ + SoilH_2_O + Temp | 166 | 6 | 449.23 | 9.78 | 0 | 0.42 |
| NH_4_^+^ + SoilH_2_O + Temp + Precip | 166 | 7 | 453.71 | 14.26 | 0.01 | 0.42 |
| NO_3_^-^ + NH_4_^+^ + VolH_2_O + Temp + Precip | 166 | 8 | 457.82 | 18.37 | 0.02 | 0.42 |
| **FERTILIZATION** |  |  |  |  |  |  |
| NO_3_^-^+ NH_4_^+^ | 198 | 5 | 427.91 | 0.00 | 0.23 | 0.61 |
| NO_3_^-^ | 198 | 4 | 428.61 | 0.69 | 0.23 | 0.61 |
| NO_3_^-^ + NH_4_^+^+ Precip | 198 | 6 | 434.24 | 6.33 | 0.23 | 0.61 |
| NO_3_^-^ + NH_4_^+^+ Temp + Precip | 198 | 7 | 438.59 | 10.68 | 0.23 | 0.61 |
| NO_3_^-^ + NH_4_^+^ + Temp + SoilH_2_O + Precip | 198 | 8 | 444.86 | 16.95 | 0.18 | 0.61 |
| **ALL** |  |  |  |  |  |  |
| NO3+NH4 | 487 | 5 | 1157.02 | 0.00 | 0.31 | 0.42 |
| NO3+NH4+Precip | 487 | 6 | 1163.58 | 6.55 | 0.31 | 0.42 |
| NO3+NH4+SoilH2O+Precip | 487 | 7 | 1170.43 | 13.41 | 0.31 | 0.42 |
| NO3 | 487 | 4 | 1183.68 | 26.66 | 0.25 | 0.38 |

**Table S5: Extractable ammonium and total inorganic nitrogen (TIN) in soils (0 to 400 cm). Standard deviations are shown in parentheses.**

| Treatment | NH_4_^+^ kg ha^-1^ | NH_4_^+^ kg ha^-1^  Trt - CTL | TIN kg ha^-1^ | TIN kg ha^-1^  Trt - CTL |
| --- | --- | --- | --- | --- |
| 0 | 10.4  (3.6) | N.A. | 228  (21.6) | N.A. |
| 80 | 7.3  (4.0) | -3.1  (2.4) | 292  (87.0) | 64.4  (90.5) |
| 120 | 4.6  (7.8) | -5.8  (10.5) | 332  (71.6) | 104  (92.0) |
| 160 | 2.7  (4.3) | -7.7  (6.6) | 385  (110) | 157  (128) |
| 200 | 12.5  (18.1) | 2.2  (22) | 439  (172) | 211  (191) |

**Table S6.** **Average and standard deviation of soil nutrient content for all treatments on all dates in top 10cm of soil.**

| **Date** | **Treatment** | **Mean NH_4_-N (kg ha^-1^)** | **Std. dev NH_4_-N** | **Mean NO_3_-N (kg ha^-1^)** | **Std. dev NO_3_-N** |
| --- | --- | --- | --- | --- | --- |
| 19-Nov | Soybean | 1.66 | 0.48 | 4.06 | 2.06 |
| 3-Dec | Soybean | 3.86 | 0.76 | 0.25 | 0.21 |
| 17-Dec | Soybean | 3.06 | 1.32 | 0.11 | 0.07 |
| 8-Jan | Soybean | 2.26 | 1.19 | 0.95 | 0.58 |
| 29-Jan | Soybean | 1.58 | 1.62 | -0.75 | 0.42 |
| 30-Jan | Maize Planting |  |  |  |  |
|  | 0 | 2.88 | 1.67 | 1.30 | 0.63 |
|  | 80 | 3.15 | 0.95 | 1.59 | 0.69 |
|  | 120 | 3.25 | 0.75 | 3.14 | 2.06 |
|  | 160 | 7.85 | 11.03 | 2.04 | 0.79 |
|  | 200 | 4.02 | 0.49 | 2.46 | 1.84 |
| 31-Jan |  |  |  |  |  |
|  | 0 | 5.14 | 1.02 | 2.92 | 1.40 |
|  | 80 | 5.21 | 2.70 | 3.10 | 1.11 |
|  | 120 | 4.38 | 2.15 | 5.79 | 5.09 |
|  | 160 | 5.19 | 2.81 | 4.57 | 3.12 |
|  | 200 | 3.96 | 1.06 | 4.05 | 2.26 |
| 1-Feb |  |  |  |  |  |
|  | 0 | 8.13 | 2.30 | 1.93 | 2.27 |
|  | 80 | 8.87 | 4.17 | 1.26 | 0.89 |
|  | 120 | 8.58 | 4.89 | 1.81 | 1.53 |
|  | 160 | 8.20 | 3.88 | 1.12 | 0.83 |
|  | 200 | 11.59 | 4.72 | 1.47 | 0.99 |
| 2-Feb |  |  |  |  |  |
|  | 0 | 2.24 | 1.23 | 2.02 | 1.10 |
|  | 80 | 3.47 | 0.71 | 3.66 | 1.58 |
|  | 200 | 4.14 | 1.19 | 4.15 | 1.15 |
| 3-Feb |  |  |  |  |  |
|  | 0 | 2.96 | 1.10 | 2.75 | 1.46 |
|  | 80 | 8.93 | 12.35 | 3.38 | 1.30 |
|  | 200 | 3.80 | 2.10 | 3.38 | 2.65 |
| 4-Feb |  |  |  |  |  |
|  | 0 | 2.09 | 0.62 | 1.60 | 1.05 |
|  | 80 | 1.94 | 0.85 | 1.31 | 1.10 |
|  | 120 | 7.10 | 3.23 | 1.95 | 1.13 |
|  | 160 | 2.60 | 1.15 | 2.19 | 1.21 |
|  | 200 | 3.78 | 1.66 | 4.31 | 2.72 |
| 5-Feb |  |  |  |  |  |
|  | 0 | 6.64 | 2.09 | 2.13 | 0.34 |
|  | 80 | 6.59 | 2.36 | 1.54 | 0.70 |
|  | 200 | 8.44 | 3.05 | 2.69 | 1.65 |
| 12-Feb |  |  |  |  |  |
|  | 0 | 2.66 | 0.87 | 1.37 | 1.28 |
|  | 80 | 3.06 | 0.99 | 2.30 | 1.49 |
|  | 120 | 2.14 | 1.20 | 1.33 | 0.80 |
|  | 160 | 2.93 | 0.42 | 3.54 | 1.67 |
|  | 200 | 3.67 | 1.37 | 3.89 | 1.32 |
| 20-Feb | Second Fertilization |  |  |  |  |
|  | 0 | 2.63 | 1.25 | 0.29 | 0.46 |
|  | 80 | 47.41 | 40.21 | 1.76 | 1.16 |
|  | 120 | 33.51 | 35.66 | 0.96 | 0.51 |
|  | 160 | 36.63 | 26.98 | 1.30 | 0.86 |
|  | 200 | 79.29 | 29.32 | 1.93 | 0.94 |
| 21-Feb |  |  |  |  |  |
|  | 0 | 3.63 | 0.83 | 0.82 | 0.22 |
|  | 80 | 133.27 | 39.18 | 6.21 | 2.68 |
|  | 120 | 69.41 | 15.28 | 4.32 | 1.82 |
|  | 160 | 138.51 | 107.43 | 6.50 | 1.01 |
|  | 200 | 153.62 | 58.43 | 6.55 | 3.00 |
| 22-Feb |  |  |  |  |  |
|  | 0 | 2.65 | 0.98 | 1.17 | 0.97 |
|  | 80 | 44.29 | 34.83 | 12.04 | 5.43 |
|  | 120 | 91.63 | 31.28 | 14.41 | 4.85 |
|  | 160 | 90.14 | 64.37 | 9.79 | 3.53 |
|  | 200 | 144.21 | 50.38 | 15.02 | 2.55 |
| 23-Feb |  |  |  |  |  |
|  | 0 | 2.79 | 0.52 | 1.33 | 1.56 |
|  | 80 | 45.76 | 18.07 | 14.91 | 10.71 |
|  | 120 | 51.39 | 33.51 | 13.58 | 2.02 |
|  | 160 | 67.08 | 21.01 | 17.80 | 2.51 |
|  | 200 | 125.22 | 79.08 | 14.72 | 3.66 |
| 24-Feb |  |  |  |  |  |
|  | 0 | 3.12 | 0.92 | 1.77 | 0.93 |
|  | 80 | 28.88 | 26.66 | 21.79 | 6.20 |
|  | 120 | 36.61 | 37.06 | 19.96 | 10.99 |
|  | 160 | 38.69 | 23.93 | 24.84 | 12.14 |
|  | 200 | 82.97 | 40.30 | 31.04 | 7.38 |
| 25-Feb |  |  |  |  |  |
|  | 0 | 3.91 | 1.46 | 1.22 | 1.06 |
|  | 80 | 12.61 | 7.69 | 15.28 | 5.51 |
|  | 120 | 12.90 | 9.48 | 28.89 | 5.71 |
|  | 160 | 24.94 | 16.23 | 33.04 | 18.50 |
|  | 200 | 45.28 | 35.15 | 30.27 | 7.12 |
| 26-Feb |  |  |  |  |  |
|  | 0 | 2.51 | 0.45 | 1.92 | 0.82 |
|  | 80 | 4.33 | 3.49 | 20.73 | 10.26 |
|  | 120 | 13.51 | 13.55 | 39.06 | 14.43 |
|  | 160 | 15.80 | 22.85 | 38.04 | 17.63 |
|  | 200 | 36.98 | 26.10 | 51.79 | 23.61 |
| 5-Mar |  |  |  |  |  |
|  | 0 | 3.54 | 1.58 | 0.27 | 0.16 |
|  | 80 | 3.47 | 1.20 | 3.91 | 2.95 |
|  | 120 | 2.60 | 0.96 | 3.83 | 3.80 |
|  | 160 | 4.02 | 1.02 | 2.63 | 1.05 |
|  | 200 | 9.86 | 8.63 | 11.05 | 11.78 |
| 12-Mar |  |  |  |  |  |
|  | 0 | 2.08 | 0.64 | 0.41 | 0.37 |
|  | 80 | 2.33 | 0.68 | 1.37 | 2.12 |
|  | 120 | 2.48 | 0.80 | 2.54 | 2.03 |
|  | 160 | 3.57 | 1.68 | 10.52 | 20.20 |
|  | 200 | 2.40 | 0.55 | 2.84 | 1.28 |
| 19-Mar |  |  |  |  |  |
|  | 0 | 4.49 | 1.27 | 0.20 | 0.24 |
|  | 80 | 3.80 | 1.20 | 0.36 | 0.39 |
|  | 120 | 3.38 | 1.29 | 1.80 | 2.22 |
|  | 160 | 4.56 | 1.72 | 2.95 | 4.72 |
|  | 200 | 3.94 | 0.93 | 2.87 | 1.51 |
| 26-Mar |  |  |  |  |  |
|  | 0 | 4.26 | 0.66 | 0.06 | 0.11 |
|  | 80 | 3.59 | 0.82 | 0.13 | 0.07 |
|  | 120 | 4.08 | 0.84 | 0.76 | 1.33 |
|  | 160 | 3.73 | 1.10 | 0.70 | 1.04 |
|  | 200 | 3.54 | 0.64 | 2.38 | 2.06 |
| 9-Apr |  |  |  |  |  |
|  | 0 | 3.24 | 1.35 | 0.01 | 0.18 |
|  | 80 | 2.60 | 0.98 | 0.13 | 0.27 |
|  | 120 | 2.94 | 0.43 | 0.14 | 0.11 |
|  | 160 | 1.99 | 1.39 | 0.75 | 0.57 |
|  | 200 | 1.45 | 2.05 | 0.44 | 0.80 |
| 23-Apr |  |  |  |  |  |
|  | 0 | 1.92 | 1.23 | 0.08 | 0.14 |
|  | 80 | 3.34 | 0.82 | 0.49 | 0.50 |
|  | 120 | 2.37 | 1.03 | 0.10 | 0.09 |
|  | 160 | 2.42 | 0.27 | 0.87 | 0.90 |
|  | 200 | 2.82 | 0.96 | 0.50 | 0.24 |


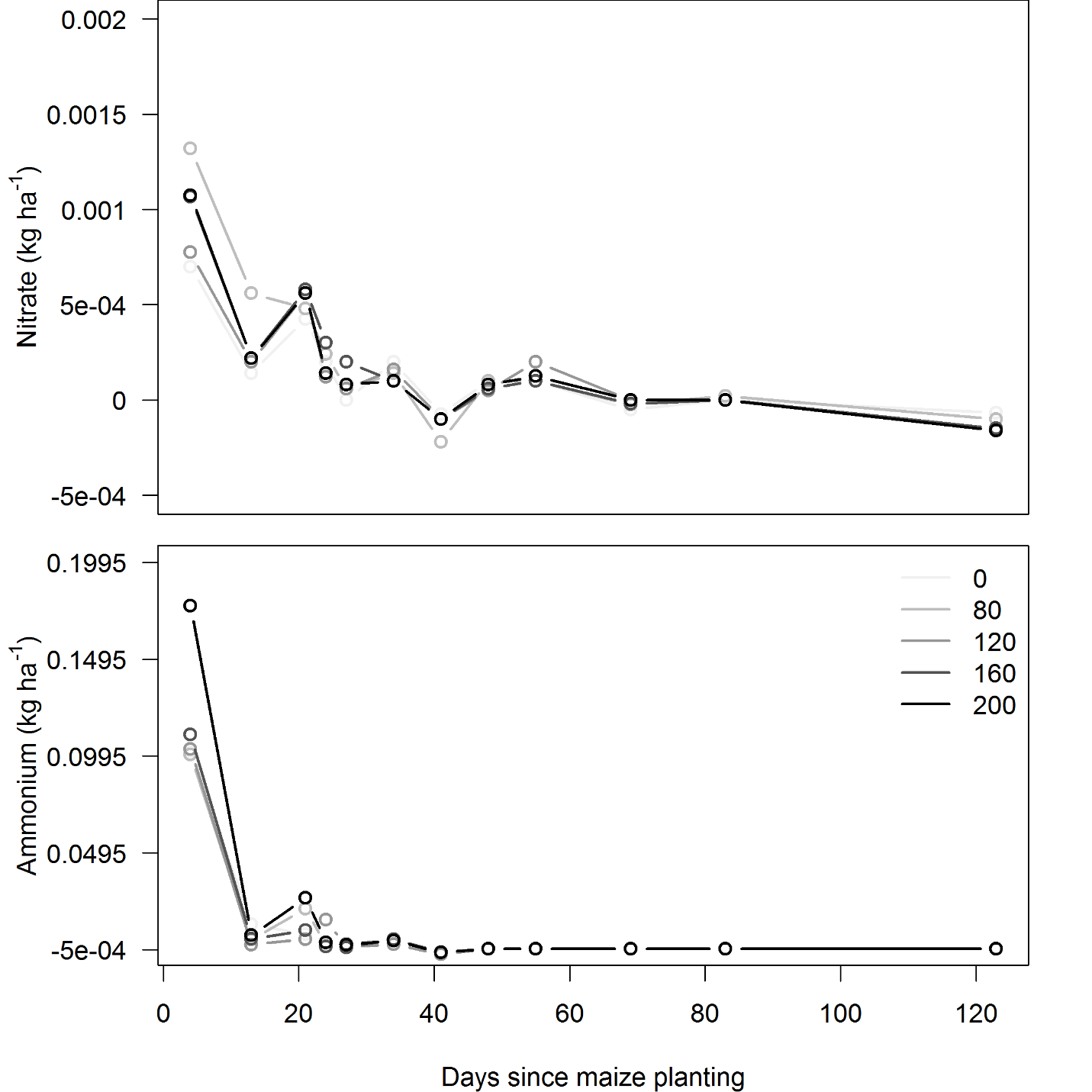


**Fig. S1**. Calculated daily loads of nitrate and ammonium from tension lysimeters installed below experimental plots at depth of 150 cm.


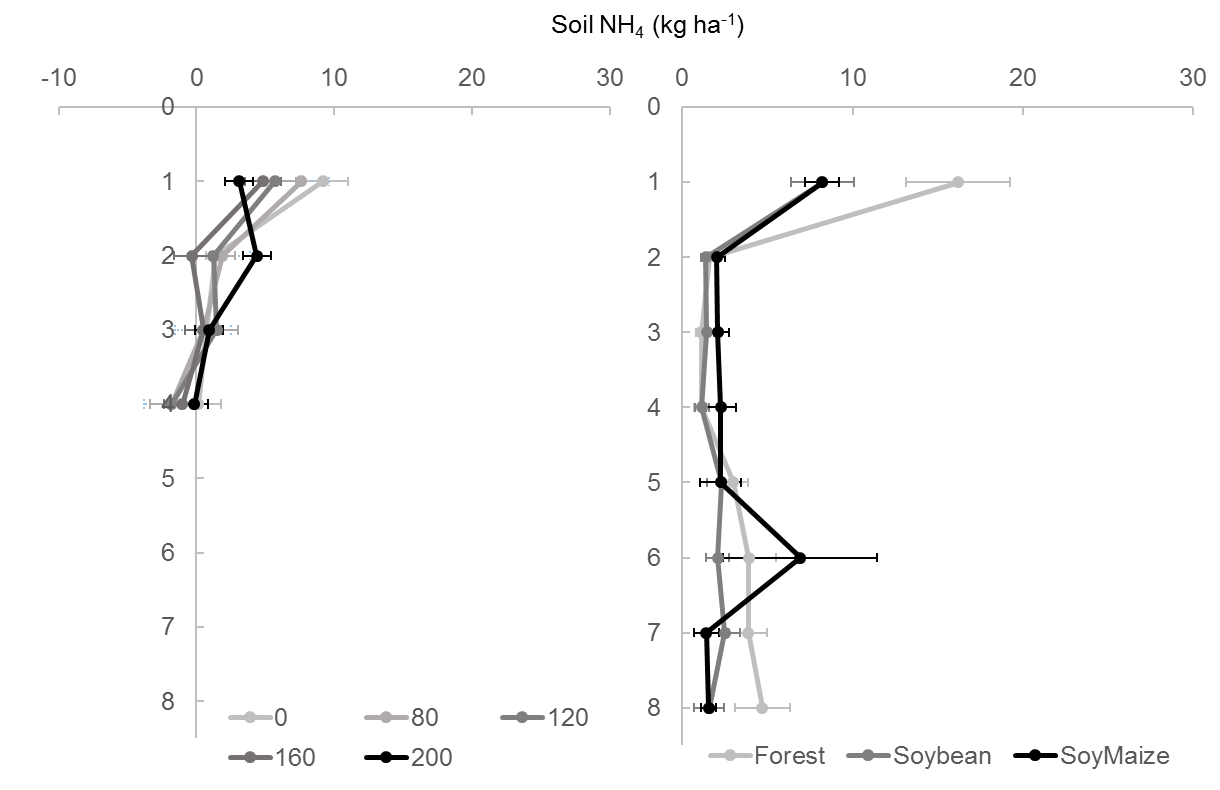


**Fig. S2.** Extractable ammonium in deep soils below experimental fertilization plots (0-4m depth).


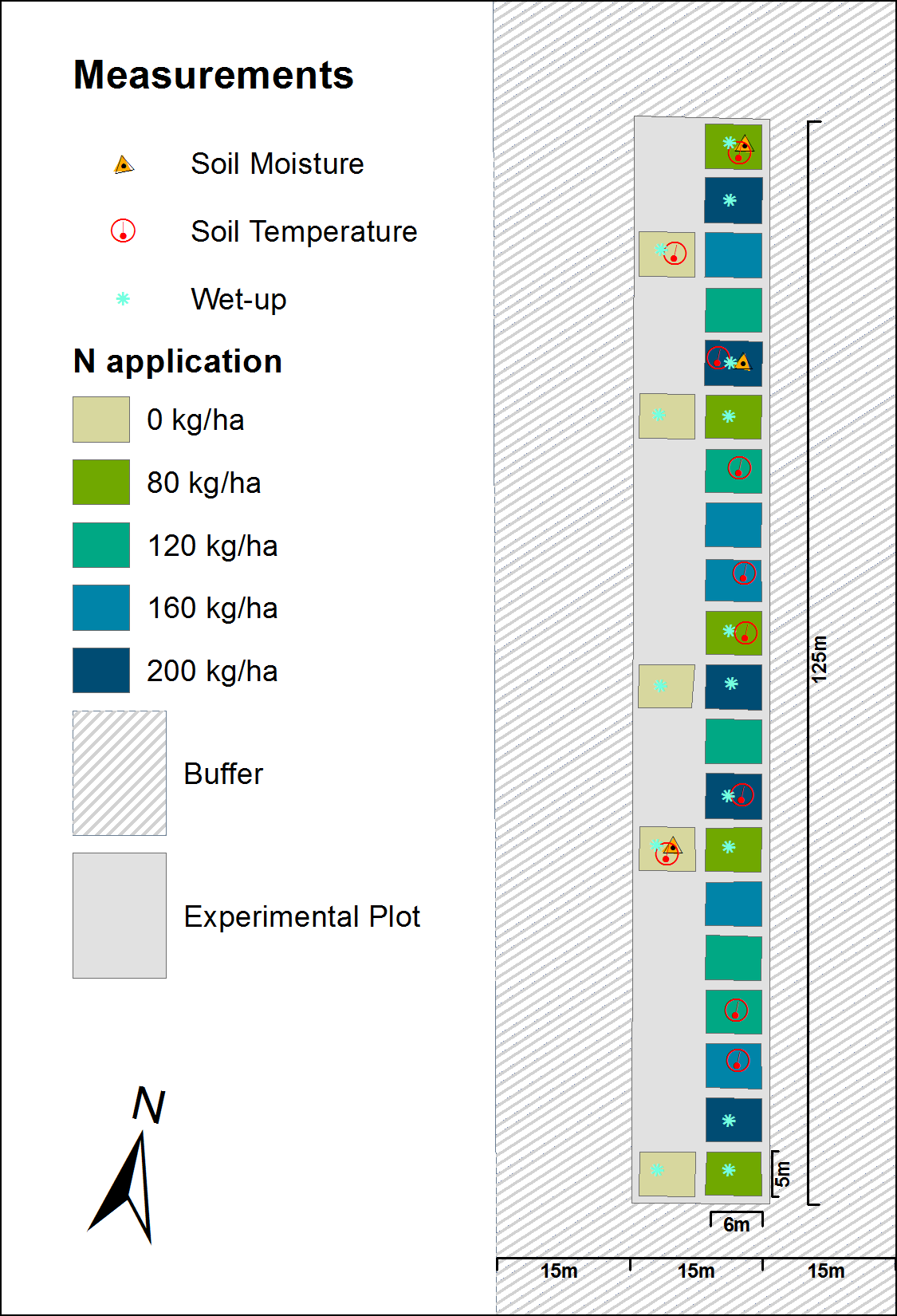


**Fig. S3**. Design of fertilization experiment.


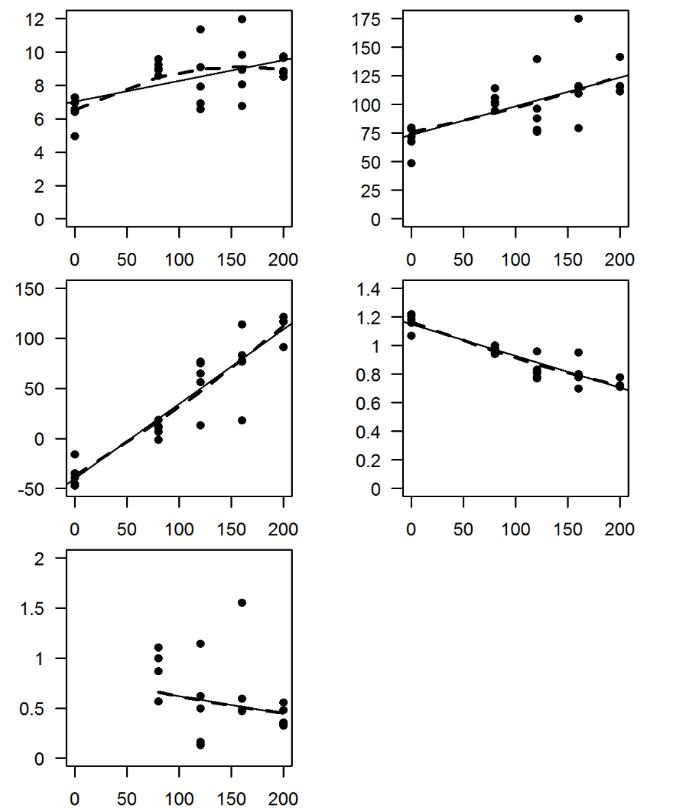


Grain Yield (Mg ha^-1^)

Grain N (kg ha^-1^)

N Surplus (kg ha^-1^)

N Use Efficiency

N Recovery Efficiency

(kg kg^-1^)

Fertilizer Treatment

**Fig. S4.** Response of Grain yield, grain N content, N surplus, N use efficiency, and N recovery efficiency to fertilizer application. Linear model shown as solid line and second best model by AICc (Table S2) shown as dashed line.


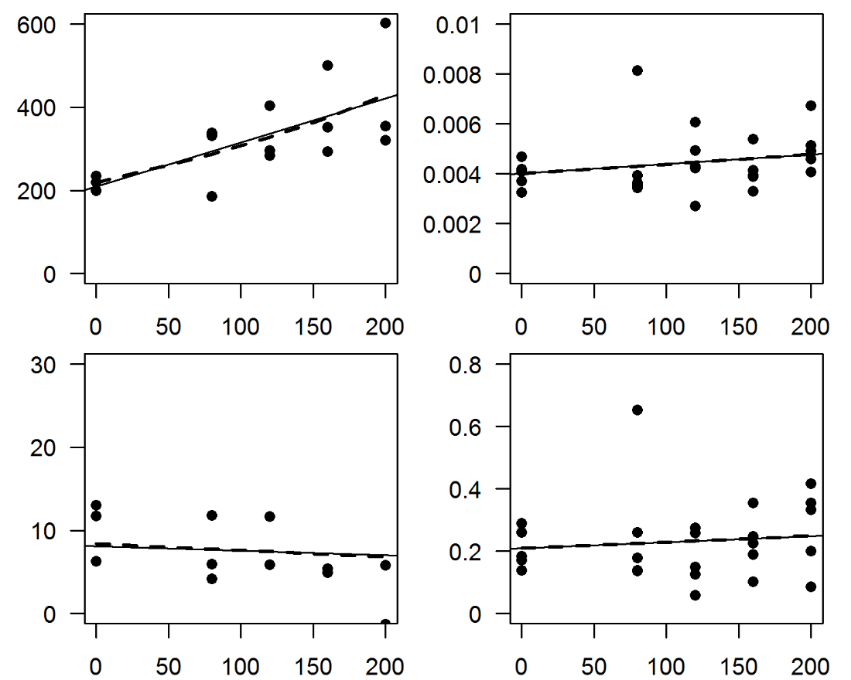


Extractable N (0-4m; kg ha^-1^)

Lysimeter N Leached (; kg ha^-1^)

NO_3_^-^-N

NO_3_^-^-N

NH_4_^+^-N

NH_4_^+^-N

Fertilizer Treatment

**Fig. S5.** Response of extractable nitrate and ammonium from 0 to 400 cm and nitrate and ammonium concentrations in lysimeters at 150 cm to fertilizer application. Solid line shows linear model fit and dashed lines shows exponential model fit.


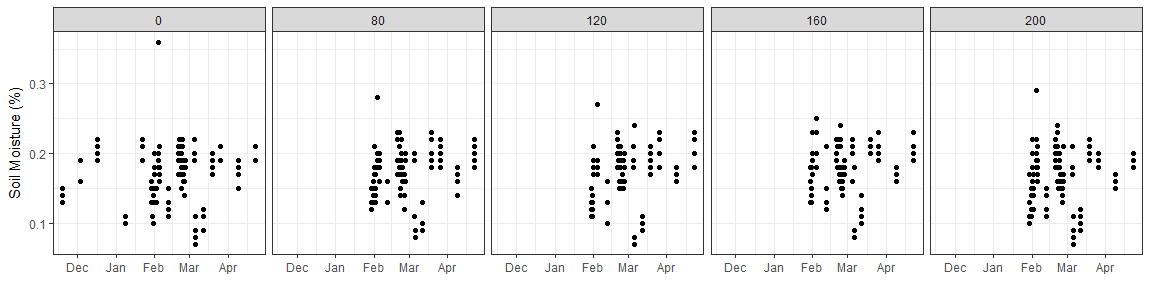


**Figure S6.** Soil gravimetric moisture content in top 0-10cm for each of the maize fertilization experiment treatments (“0”, “80”, “120”, “160”, and “200”). Samples were measured on the same soil samples taken for nutrient content. Pre-maize values are shown only for control treatment (“0”).
